# It is time for a unified definition of native vertebral osteomyelitis: a framework proposal

**DOI:** 10.5194/jbji-9-173-2024

**Published:** 2024-06-24

**Authors:** Francesco Petri, Omar Mahmoud, Said El Zein, Ahmad Nassr, Brett A. Freedman, Jared T. Verdoorn, Aaron J. Tande, Elie F. Berbari

**Affiliations:** 1 Division of Public Health, Infectious Diseases and Occupational Medicine, Department of Medicine, Mayo Clinic College of Medicine and Science, Mayo Clinic, Rochester, 55905 MN, USA; 2 Department of Infectious Diseases, ASST Fatebenefratelli Sacco, “L. Sacco” University Hospital, 20157 Milan, Italy; 3 Department of Orthopedic Surgery, Mayo Clinic, Rochester, 55905 MN, USA; 4 Department of Radiology, Mayo Clinic, Rochester, 55905 MN, USA

## Abstract

In recent years, there has been a notable increase in research output on native vertebral osteomyelitis (NVO), coinciding with a rise in its incidence. However, clinical outcomes remain poor, due to frequent relapse and long-term sequelae. Additionally, the lack of a standardized definition and the use of various synonyms to describe this condition further complicate the clinical understanding and management of NVO. We propose a new framework to integrate the primary diagnostic tools at our disposal. These collectively fall into three main domains: clinical, radiological, and direct evidence. Moreover, they and can be divided into seven main categories: (a) clinical features, (b) inflammatory biomarkers, (c) imaging techniques, microbiologic evidence from (d) blood cultures and (e) invasive techniques, (f) histopathology, and (g) empirical evidence of improvement following the initiation of antimicrobial therapy. We provide a review on the evolution of these techniques, explaining why no single method is intrinsically sufficient to formulate an NVO diagnosis. Therefore, we argue for a consensus-driven, multi-domain approach to establish a comprehensive and universally accepted definition of NVO to enhance research comparability, reproducibility, and epidemiological tracking. Ongoing research effort is needed to refine these criteria further, emphasizing collaboration among experts through a Delphi method to achieve a standardized definition. This effort aims to streamline research, expedite accurate diagnoses, optimize diagnostic tools, and guide patient care effectively.

## 1 Background

Native vertebral osteomyelitis (NVO) accounts for ∼3 % to 5 % of all osteomyelitis cases (Sobottke et al., 2008). Its incidence has progressively risen in the United States in recent years, from 2.9 to 5.4 cases per 100 000 people (Issa et al., 2018). The rise in its incidence may be attributed to the simultaneous growth of an older population with comorbidities. Clinical outcomes continue to be poor, as a significant subset of patients with NVO experience relapses, in approximately 15 %–31 % of cases (Thavarajasingam et al., 2023), and long-term sequelae (including residual neurological deficits), in about 16 %–32 % of patients (Mylona et al., 2009; Gupta et al., 2014).

Several vital topics continue to dominate the discourse surrounding NVO, reflecting ongoing advancements and challenges in the field. Notably, the optimal diagnostic strategies for NVO remain a subject of intense investigation, with emerging technologies such as advanced imaging modalities and molecular diagnostics sparking interest. The increasing recognition of atypical pathogens has broadened the spectrum of causative agents (Maamari et al., 2022). Furthermore, there is a growing emphasis on the duration, choice, and route of antimicrobial therapy and the role of surgical intervention in specific cases, reflecting a nuanced understanding of the disease course and patient outcomes. The intersection of NVO in immunocompromised hosts, such as those with diabetes mellitus or immunosuppressive therapies, is another expanding area of research, shedding light on unique challenges and considerations in these populations. Addressing these topics not only refines our understanding of NVO but also paves the way for more targeted and practical approaches to diagnosing, treating, and managing this complex infectious condition.

The progressive introduction of novel molecular and radiographic techniques into clinical practice over the past 2 decades has enhanced our diagnostic capabilities for NVO. These advancements have contributed to a substantial increase in research output, with over 8400 articles (Petri, 2024) on NVO and its synonyms having been indexed in the literature in the last 20 years. However, despite the increase in scholarly attention, the landscape of these articles, predominantly retrospective in design, has relied on a patchwork of definitions, significantly impeding research comparability, reproducibility, and epidemiological tracking of NVO.

This article explores the roots of this definitional ambiguity and advocates for a consensus-driven, multidisciplinary approach to establishing a comprehensive and universally accepted definition of NVO. We believe that a uniform definition will foster scientific advancement and ultimately enhance patient care.

**Figure 1:**
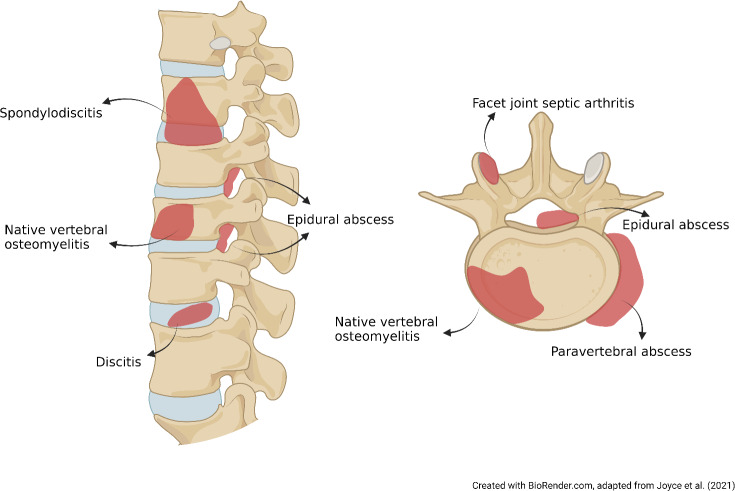
Different localizations and visual definitions of NVO syndrome.

## 2 A closer look at terminology and classification

`Spondylodiscitis”, “NVO”, “pyogenic vertebral osteomyelitis”, “spondylitis”, “discitis-osteomyelitis”, and “disc-space infection” are a few terms used interchangeably to describe the same clinical condition. The variability in these synonyms highlights the need for standard, precise terminology. Classical logic affirmed that names are consequences of the things that they denote. For example, spondylodiscitis combines aspects of “spondylitis” (inflammation of the vertebra) and “discitis” or “diskitis” (inflammation of the spinal disk). However, it remains unclear if this term fully captures the infection of the vertebral bone and its specific etiology, whether infective or inflammatory (Fig. 1). Moreover, facet joint septic arthritis and postsurgical infections are considered to be a separate nosological entity from NVO due to their different pathogenic mechanism and prognosis (Babic and Simpfendorfer, 2017; Nasto et al., 2012).

**Figure 2:**
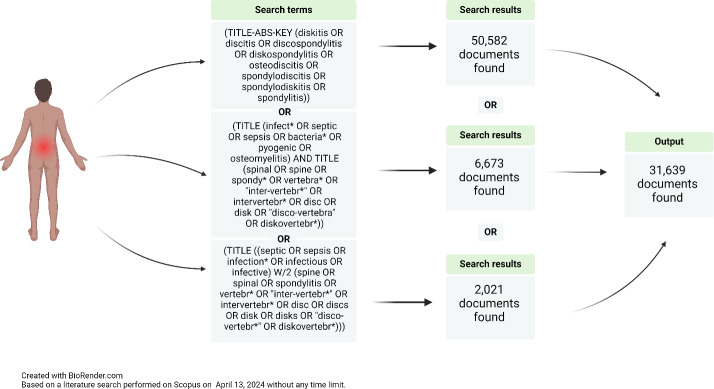
Example of a search strategy conducted by a specialized medical librarian looking for NVO papers.

This variation in closely related terms complicates literature review and synthesis, as relevant studies may be overlooked despite thorough search strategies (Fig. 2). Moreover, a comprehensive classification based on high-quality evidence by etiology (pyogenic, brucella, fungal, mycobacterial, parasitical, or culture-negative), pathophysiology (e.g., hematogenous seeding, direct inoculation at the time of spinal surgery, or contiguous spread from an infection in the adjacent structures; Zimmerli, 2010), and natural history (e.g., acute, subacute, or chronic) is lacking, as literature relies primarily on observational studies and case series, due to the relative rarity of the condition. Only one randomized controlled trial focused on pyogenic vertebral osteomyelitis (Bernard et al., 2015) has been published to date, and clinical practice predominantly references the 2015 Infectious Disease Society of America (IDSA) guidelines on NVO (Berbari et al., 2015).

## 3 The blind men and the elephant

The tale of the blind men and the elephant provides a fitting analogy for the diagnostic challenges in NVO, as it also did for infective endocarditis (IE) in the past (Fowler, 2023). In this story, each blind man touches a different part of the elephant, leading to varied and incomplete interpretations of the whole animal. Similarly, in diagnosing NVO, clinicians must rely on various fragmented information sources.

**Figure 3:**
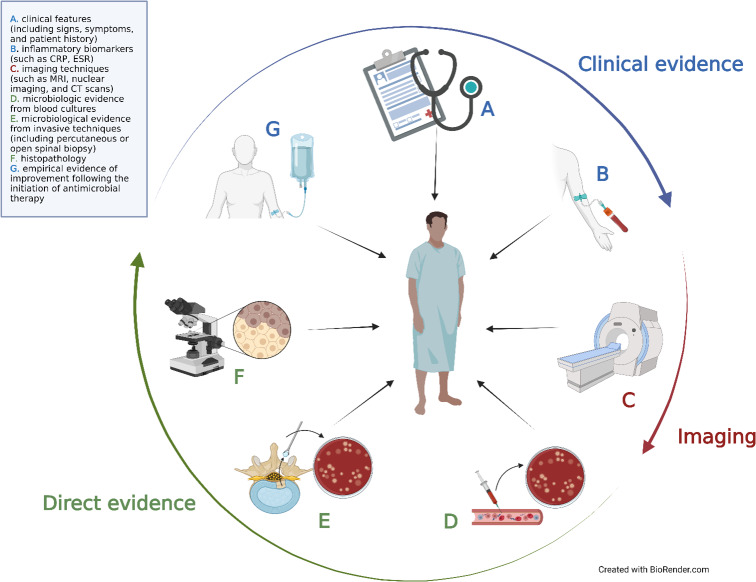
Seven main categories were grouped into three domains proposed to establish NVO diagnosis.

The challenge in diagnosing NVO lies in integrating these diverse pieces of information into a cohesive understanding of the syndrome. The primary diagnostic tools at our disposal can be divided into seven main categories: (a) clinical features (including signs, symptoms, and patient history), (b) inflammatory biomarkers (such as C-reactive protein, CRP, and erythrocyte sedimentation rate, ESR), (c) imaging techniques (such as plain film X-rays; magnetic resonance imaging, MRI; nuclear imaging; and computed tomography, CT, scans), microbiologic evidence from (d) blood cultures and (e) invasive techniques (including percutaneous or open spinal biopsy), (f) histopathology, and (g) empirical evidence of improvement following the initiation of antimicrobial therapy. These modalities collectively fall into three main domains: clinical, radiological, and direct evidence (Fig. 3).

**Figure 4:**
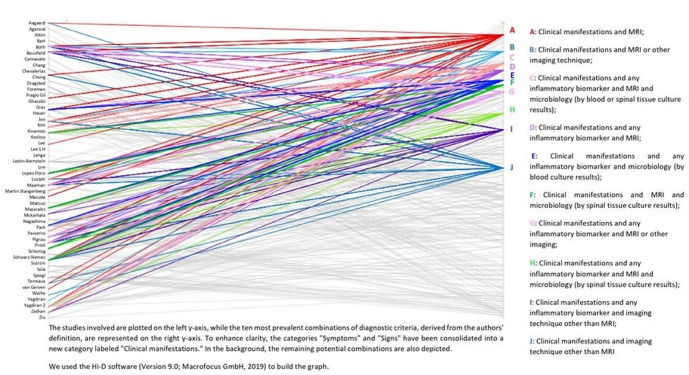
Parallel coordinates plot of the 10 most prevalent potential unique combinations of diagnostic criteria for NVO used by the authors of 50 published cohort studies with at least 50 NVO cases (Petri, 2024).

As there are currently no accepted diagnostic standards for NVO, authors often diagnose NVO using a somewhat arbitrary combination of these standards (Fig. 4).

### 3.1 Clinical evidence alone might not be enough to diagnose NVO

The clinical domain involves a patient's history and physical examination. Based on the 2015 IDSA guidelines (Berbari et al., 2015), clinicians should consider an NVO diagnosis in patients with new or worsening back/neck pain, fever, or bloodstream infection/endocarditis. Fever and neurological symptoms with or without back pain or recent *Staphylococcus aureus* bloodstream infection (BSI) with new localized neck/back pain are also grounds for suspecting NVO. Recent French guidelines for spondylodiscitis (Lacasse et al., 2023) propose considering this syndrome if there is recent or worsening febrile spinal pain, spinal pain with bacteraemia and/or elevated CRP, or fever and/or spinal pain and/or elevated CRP and/or scarring disorder following a spinal procedure. Spinal pain with red flags, e.g., those proposed by Yusuf et al. (2019), should prompt a systematic search for NVO syndrome.

The classical triad of fever, back pain, and neurological dysfunction has been described; however, only a tiny percentage of patients present with all three symptoms. Relying solely on this triad may result in a high burden of missed cases or delayed diagnosis, leading to poor outcomes. Davis et al. (2004) reported the classic triad to be 8 % sensitive and 99 % specific for spinal epidural abscess (Davis et al., 2004). In most cohort studies, the most prevalent reported symptom is back pain (Yusuf et al., 2019); however, back pain is usually benign, with an 80 % prevalence in the general population (Rubin, 2007). Relying solely on this symptom for NVO diagnosis is, therefore, unhelpful. Fever often accompanies infectious conditions but is present in only about 50 % of NVO patients (Mylona et al., 2009); thus, its absence cannot rule out NVO. History can guide clinicians; older age, intervertebral disk degeneration, prior infections (especially urinary tract infections and skin and soft-tissue infections), injection-drug use, bacterial endocarditis, previous surgery or corticosteroid injections, immunocompromised status, diabetes, and chronic kidney failure are common risk factors.

### 3.2 Imaging evidence alone might not be enough to diagnose NVO

Various imaging modalities can be used to assess NVO. Moreover, imaging is also helpful in guiding management, e.g., planning for invasive diagnostics, need for surgery, or assessing the presence of abscesses that may require drainage. Historically, plain radiographs were initially recommended for cost-effectiveness and to rule out alternative causes of back pain. However, their sensitivity, particularly in the early stages of infection, is limited (Maamari et al., 2023). Serial plain films and inflammatory biomarkers still have a role in postoperative monitoring, reserving CT scans and/or MRI with gadolinium in case of concern for progression. MRI swiftly became the preferred modality, with high sensitivity (96 %), specificity (92 %), and accuracy (94 %) in diagnosing vertebral osteomyelitis, according to early studies (Modic et al., 1985), while its performance appeared to more equivocal in more recent works (Smids et al., 2017). The use of follow-up MRI in selected patients has been discussed elsewhere (Kowalski et al., 2006, 2007). While computed tomography (CT) and nuclear imaging techniques like ^67^Ga, ^99m^TC, and ^111^In have been explored, prior reviews have suggested inferiority to MRI in most situations (Zimmerli, 2010), mainly due to poorer anatomic resolution.

Recent advancements, including increased utilization of single-photon-emission CT with scintigraphy and positron-emission tomographic (PET) imaging, have reignited interest in their efficacy in NVO diagnosis. A recent systematic review by Maamari et al. (2023) found that positron emission tomography–computed tomography (PET/CT) and MRI exhibit similar sensitivities (93 % and 90 %, respectively), with PET/CT having slightly better specificity (80 % vs. 72 %). However, the authors caution that these findings alone may not justify a significant shift in the imaging diagnosis approach for NVO. Notably, among nuclear techniques, ^67^Ga demonstrated a sensitivity of 95 % and a specificity of 88 %, with enhanced specificity when combined with ^99m^Tc. Moreover, PET/CT and MRI play a pivotal role in localizing *S. aureus* infection foci, even without back pain, offering valuable insights (Goodman et al., 2023).

Despite the utility of these imaging methods, distinguishing NVO from conditions with similar presentations can be challenging but is imperative due to therapy and prognosis implications. For instance, the “claw sign” observed on diffusion-weighted MRI strongly indicates Modic type-1 degenerative changes (Patel et al., 2014) rather than NVO. Inflammatory cases may manifest with clues such as multilevel involvement and sacroiliitis, suggesting a spondyloarthropathy diagnosis. Atypical radiological findings, such as a black vertebra on post-contrast T1-weighted imaging indicating early emphysematous infection, highlight the need for careful consideration and additional investigations (Park et al., 2020).

### 3.3 Microbiological evidence alone might not be enough to diagnose NVO

The success of antibiotic therapy in treating NVO heavily depends on the precise identification of the causative pathogen(s). Again, establishing a microbiological diagnosis is of high importance with respect to providing information for disease management. However, difficulty arises from the broad microbiological spectrum capable of causing NVO, making it challenging to rely solely on cultures for diagnosis. The IDSA guidelines highlight the necessity to differentiate between typical and atypical bacteria as causes of NVO (Berbari et al., 2015). Identification of typical pathogens like *S. aureus* complex, the rarer *Staphylococcus lugdunensis*, or *Brucella* species through blood cultures or serologic tests with adequate titer results may eliminate the need for further investigation if compatible imaging or syndromes are present (Berbari et al., 2015). In cases where these tests fail to confirm a microbiologic diagnosis, an image-guided biopsy, targeting fluid collections if present, and/or the intervertebral disk, the endplate, the bone, or paraspinal tissue, may become essential (Berbari et al., 2015; Husseini et al., 2021). Compatible imaging combined with the isolation of an organism from a biopsy can suffice for diagnosis. However, the effectiveness of both noninvasive and invasive conventional microbiology tests, as well as serologic tests, is traditionally considered less than ideal. Despite the lack of high-quality data, the isolation of other virulent bacteria microorganisms (i.e., streptococci, enterococci, and Enterobacteriaceae) from blood culture in the presence of a compatible clinical and radiological picture is increasingly accepted in clinical practice as confirmatory of NVO. Conversely, certain situations are common in which a positive culture result may not be conclusive. Instances such as coagulase-negative staphylococci (CoNS) positivity in blood cultures with indeterminate imaging and the presence of *Cutibacterium acnes* or *Corynebacterium* species in biopsy samples are typical examples. The literature shows the importance of interpreting these isolates, including the type of microorganism, the number of positive culture sets, the number of positive blood culture bottles within a set, the colony-forming unit (CFU) quantification, and the timing of growth (Dargère et al., 2018). All of these factors may be useful in confirming isolates as pathogenic or deeming them contaminants. When a possible contaminant, e.g., CoNS, is isolated from one or two blood culture bottles, it might be challenging to discriminate between a true pathogen or a contaminant within just one set (e.g., two bottles). Clinical judgment must be therefore used. In contrast, within two or three sets of blood culture bottles (e.g., four to six bottles), this type of bacteria can be more easily considered to be a contaminant. This evidence stresses the need to avoid solitary blood cultures (i.e., only one set or single couple of blood cultures) and to improve the overall diagnostic quality in blood culture collection and processing (Choi et al., 2017; Leyssene et al., 2011; Tokars, 2004).

Similarly, for results from biopsy, it is reasonable to consider the isolation of *C. acnes* from a single culture as clinically insignificant, especially if a longer time to positivity of cultures is demonstrated (Passerini et al., 2023). Other previous experiences have shown that it is still unclear whether having two or more positive intraoperative cultures is sufficiently specific to clearly confirm an infection caused by *C. acnes* (Bumgarner et al., 2020; Tai et al., 2023). This is especially true for hardware-associated infections and prosthetic joint infections (PJI), for which more literature is available (Boisrenoult, 2018; Parvizi et al., 2014a).

## 4 The pathway towards a reference test for diagnosis

In recent years, histopathology and cytology from disk and/or vertebral tissue have held increasing relevance, primarily due to their practicality and accuracy in diagnosing NVO. A recent analysis by Iwata et al. (2019) focused on areas with maximum inflammatory cell infiltration and the extent of neutrophil infiltration. This analysis showed that, in most of the individuals examined, the final clinical diagnosis of both mycobacterial and pyogenic spondylodiscitis was significantly correlated with the histological results in the spinal biopsies. The presence of one or more neutrophils per high-power field, on average, emerged as a specific and sensitive marker for identifying cases of pyogenic spondylodiscitis. Additionally, Riaz et al. (2022) demonstrated the value of cell counts and neutrophil differentials from aspirates from the intervertebral disk area in patients with suspected NVO as a quick and accurate test for assisting in diagnosis. These techniques pave the way towards a more comprehensive understanding of the peculiar category of culture-negative NVO or of cases where microbiological results are inconclusive.

## 5 Examples of successful definitions in infectious diseases

Crafting a consistent and universal definition for syndromes caused by infectious agents is a long-standing and intricate challenge in the field of infectious diseases. This difficulty is notably evident in the categorization of specific conditions such as IE and PJI, which have historically faced similar challenges in their classification and definition. Recent history provides a good example of a complex and successful path for the latter (Sigmund et al., 2022). Parvizi et al. (2011) aimed for a gold standard for PJI in 2011, introducing the first PJI definition based on major or minor criteria. A 2013 modification (Parvizi et al., 2014b), post International Consensus on Musculoskeletal Infection, added a threshold for minor criteria and an algorithmic diagnostic approach. In the same year, IDSA issued international diagnostic guidance (Osmon et al., 2013), significantly improving global diagnostics (Bouaziz et al., 2018). In 2018, PJI advancements prompted experts to update the MusculoSkeletal Infection Society (MSIS) criteria with an evidence-based, weight-adjusted scoring system (Parvizi et al., 2018). A 2021 European Bone and Joint Infection Society (EBJIS) and MSIS-backed project provided a concise diagnostic framework with three levels: infection unlikely, likely, or confirmed (McNally et al., 2021).

These shared struggles mainly rely on the common impossibility of establishing, with a universal grade of certainty, the causation between a microorganism and its pathogenicity, as this is generally a spectrum and not a black-and-white condition. There is not a unique noninvasive biomarker for infection, from imaging or from blood. As a result, it is necessary to perform invasive procedures with a low diagnostic performance, low specificity, and (often) inconclusive histologic results. There is a broad variability in the clinical presentation of NVO and overlap with many other conditions of different nature (inflammatory, neoplastic, and degenerative are amongst the most common). Finally, there is a relative rarity of these conditions that renders it impossible to produce high-quality evidence through prospective observational studies or randomized controlled trials with a large sample size. These factors cause an enormous variability in the diagnostic approach and management of NVO across the literature.

## 6 The need for a consensus on diagnostic criteria

The pathway to establishing a unified definition for NVO must consider different factors, similar to what has been done with PJI (McNally et al., 2021). Diagnosing most NVO infections should rely on sensitive tests to avoid the consequences of undertreatment. It is also crucial not to overdiagnose, only confirming infection when tests with high specificity validate it. The diagnostic approach should be simple, aid decision-making in the clinical setting, and use readily available tests without unnecessary repetition. The criteria should be acceptable to a broad range of clinicians, supporting conclusions with evidence. The criteria should recognize that confidence in specific tests may change with improved research and understanding of the disease, potentially altering their role over time.

A consistent definition of NVO grounded in high-quality evidence remains elusive. This inconsistency impedes comparability, may result in delays in patient care due to uncertainties in the diagnosis given the lack of criteria, and hampers advancements in NVO research.

Identifying gaps in evidence is crucial for both patient care and research advancement in the subsequent steps of defining and developing diagnostic criteria. Moreover, addressing these separate but complementary rationales can lead to varied purposes and outcomes in definitions and diagnostic criteria. This distinction must be clearly articulated throughout the development process, as emphasized by Guyatt et al. (2008) and Alonso-Coello et al. (2016). There is, therefore, a pressing need to discern universal criteria for a clinically operational definition of NVO. Such a definition can help streamline research and diagnostic criteria; expedite accurate diagnoses; optimize the timing and choice of diagnostic tools (e.g., imaging, microbiology, molecular, and laboratory tests); and guide optimal patient care regarding treatment selection, duration, and administration method. This would progress unmet research needs by ensuring the generalizability of the results and help clinical decision-making, as it would help overcome the necessity for a gold standard for diagnosis, such as histology.

With the caveats expressed above, we propose that the new framework definition of NVO might incorporate the concepts of hematogenous seeding of the culprit bacteria. This would underline the existing separation into distinct categories between (1) native and (2) hardware-associated, postsurgical, post-decubitus ulcers, post-traumatic, secondary to fistulas, radiotherapy, other contiguity mechanisms of disease, or periprocedural inoculation of pathogens. Mycobacterial and brucellar vertebral osteomyelitis should be recognized as distinct disease entities due to historical, patient-specific, and pathogen-specific factors (Bozgeyik et al., 2008; Glassman et al., 2023; Tuli, 2013; Turunc et al., 2007). Additionally, facet joint septic arthritis also warrants separate consideration (Babic and Simpfendorfer, 2017). To ensure clarity and comprehensiveness for treating physicians, we advocate for the inclusion of these spinal infections in future discussions on the framework for definitions, highlighting their unique differences and characteristics compared with NVO.

We suggest that NVO could be diagnosed after excluding possible alternative diagnoses (Maamari et al., 2022), with a combined evaluation of the following:

- clinical features, especially back/neck pain, fever and/or neurologic deficits;
- inflammatory biomarkers, such as CRP, white blood cell count (WBC), and/or ESR;
- imaging, preferably MRI, otherwise CT, PET/CT, or combined ^67^Ga and ^>99m^Tc scintigraphy (Maamari et al., 2023);
- histopathology consistent with NVO by demonstration of acute, chronic, or granulomatous inflammation and/or pathogens (Pupaibool et al., 2015);
- microbiology results from blood cultures for a known associated organism (*S. aureus*, *S. lugdunensis* species) (Berbari et al., 2015) or from staining, molecular techniques, or cultures from spinal specimens obtained from single or multiple percutaneous image-guided spinal biopsy and/or open or endoscopic surgery. More data on new culture-independent detection techniques on whole blood or tissue are needed.
- clinical and/or radiographic improvement after empiric antimicrobial therapy (Kowalski et al., 2006).

Some of these tests may also serve dual roles – e.g., beyond just supporting or confirming diagnosis: MRI may provide information on the need for and/or extent or type of surgery (Babic and Simpfendorfer, 2017), and cultures and/or molecular microbiological results are paramount in defining the best antibiotic treatment strategy.

Ongoing research is focused on a systematic review and meta-epidemiological project targeted at NVO. This initiative seeks to systematically identify and evaluate the distribution of definitions and possible combinations of criteria used for NVO diagnosis employed in the existing literature. The derived insights will play a crucial role in the subsequent implementation of a Delphi method, involving collaboration with experts to establish a consensus on a standardized NVO definition.

By disclosing these prospective steps, we underscore our commitment to transparency and acknowledge the evolving nature of scientific research. Furthermore, we recognize that disseminating these intentions may serve as an input for additional contributions. This openness to collaboration aims to foster a dynamic and cooperative environment within the scientific community.

## Data Availability

No data sets were used in this article.
